# Radial Artery Occlusion After Transradial Access for Coronary Interventions

**DOI:** 10.7759/cureus.58036

**Published:** 2024-04-11

**Authors:** Farooq Ahmad, Ikram Ullah, Sher W Khan

**Affiliations:** 1 Cardiology, Khyber Medical College/Khyber Teaching Hospital, Peshawar, PAK; 2 Interventional Cardiology, Fauji Foundation Hospital, Peshawar, PAK; 3 Cardiology, Lady Reading Hospital and Medical Teaching Institute, Peshawar, PAK; 4 Adult Cardiology, Lady Reading Hospital and Medical Teaching Institute, Peshawar, PAK

**Keywords:** coronary diagnostics, cardiology research, coronary interventions, transradial approach, radial artery occlusion

## Abstract

Background

Transradial access (TRA) is a medical procedure primarily used for percutaneous coronary interventions (PCI) and cardiac catheterization. Based on the recently published Minimizing Adverse Haemorrhagic Events by Transradial Access Site and Systemic Implementation of AngioX (MATRIX) trial, TRA is being used more frequently than transfemoral access (TFA) since it has reduced rates of bleeding and fatality. A structural complication of TRA is radial artery occlusion (RAO), which may cause temporary pain to limit TRA in the future.

Objective

This study aimed to investigate the onset and risk factors of RAO following TRA for coronary interventions.

Material and methods

An observational study was conducted at Fauji Foundation Hospital in Peshawar, Pakistan. The study included 1,680 patients recruited between April 2021 to December 2023. Fifty-eight patients were lost to follow-up, while another 95 patients did not come for a visit within the study period. The final study, therefore, included 1,527 patients.

Results

The mean age of patients was 58.09 ± 8.07 years. Patients were divided into two age groups (greater or less than 60 years). Diagnostic angiograms were completed for 955 patients, while 572 also underwent PCI. The overall RAO onset was 81 (5.3%). There was a significantly higher RAO onset in patients over 60 years old (7.1 vs 3.8%, p = 0.003).

Conclusion

Overall, the risk of RAO is low following TRA. The risk of RAO is significantly higher in people aged over 60 years.

## Introduction

Transradial access (TRA), or transradial angiography or transradial catheterization, is a diagnostic procedure for various cardiac disorders. During the procedure, a catheter is inserted into a radial artery. This procedure was first introduced by Campeau et al. in 1989. Numerous studies have shown that TRA is beneficial since it maintains a high procedural success rate, a comparatively low possibility of access site bleeding, and high patient satisfaction [1]. Over the past decade, TRA has become much more common for all types of acute coronary attacks and immediate coronary procedures. Research indicates that TRA is associated with a notable decrease in access site bleeding issues and increased survival rates for patients receiving percutaneous coronary interventions (PCI) for acute coronary syndrome [2]. TRA can result in hematoma or perforation, but the radial artery spasm and radial artery occlusion (RAO) rates are low.

RAO is one of the complications after TRA that occurs in 1-10% of patients. Hand ischemia, although rare, is a severe problem that can occur after RAO [3] and makes it impossible to use the radial artery for coronary artery bypass grafts or arteriovenous fistula formation in the future. It has been shown that the risk of RAO can be lowered by using perfect hemostasis, a higher amount of anticoagulation, and faster compression after surgery [4,5]. Before a patient is discharged, their radial artery should be checked to ensure it is open. New research and methods are needed to identify how to reduce the risk of RAO [6]. A previous study examined the risk factors for RAO, finding that women and patients with diabetes are more likely to have acute RAO. The long time of high-pressure compression hemostasis after surgery, the amount of heparin used in the process, the type and size of the sheath, and the compression time after surgery are all risk factors for RAO [7].

This study aimed to determine RAO risk factors following TRA in coronary interventions. The risk factors of RAO resulting from TRA in Pakistani patients, especially in the province of KPK, had not been studied in detail before. Thus, the present study aims to fill this gap in the literature.

## Materials and methods

Study design

This study used a prospective cross-sectional approach. The study was conducted at Fauji Foundation Hospital in Peshawar, Pakistan. Ethical approval to conduct the study was obtained from Fauji Foundation Hospital (reference 6039/Adm). All patients were also informed about the purpose of the research and consented to participate. The patients’ data were kept confidential as their personal information was not shared with anyone other than the researchers.

Inclusion and exclusion criteria

Patients of any age and gender undergoing coronary diagnostic and therapeutic procedures through a TRA at Fauji Foundation Hospital in Peshawar were included in the study. Patients with a poor pulse, who switched to transfemoral access (TFA), whose sheath was not removed immediately after the procedure (as it can result in loss of radial pulsation which can be a bias), or with a history of previous transradial interventions were excluded from the study. All procedures were done by an interventional cardiologist, and it included all single, double, and triple vessel PCI.

Data collection

A total of 1,680 patients were included in the study, which took place between April 2021 and December 2023. Fifty-eight patients were lost to follow-up, while another 95 patients did not come for a visit within the study time. The final analyses, therefore, included data from 1,527 patients. All patients received unfractionated heparin (2500 IU) and 200 mcg of nitroglycerin following a 6F-sheath insertion. The sheath was removed immediately after the procedure, and manual pressure followed by band ligation was applied for hemostasis. Patients’ radial artery patency was reviewed by the radial pulse by a consultant cardiologist two weeks after the index procedure. Patients without a palpable pulse were labeled as having RAO. The patients were assessed in a clinical setup without the use of any gadgets such as Doppler. Thus, assessing vessel patency was the most accurate and convenient method.

Statistical analysis

Data were analyzed using the statistical analysis program Statistical Product and Service Solutions (SPSS, version 23; IBM SPSS Statistics for Windows, Armonk, NY). Frequencies and percentages were recorded for qualitative variables, including gender, cardiovascular risk factors, medications, and RAO. Means and standard deviations were calculated for age and BMI. A chi-square test was applied to find the relationship between the onset of RAO and age, gender, medical procedure, and co-morbidities. A p-value less than 0.05 was considered significant.

## Results

The mean age of the study population was 58.09 ± 8.07 years. There were 580 (38%) females in this study. The most common cardiovascular risk factor was hypertension 1,236 (81%), followed by diabetes mellitus (363, 23.8%) and dyslipidemia (320, 21%). There were 626 (41%) patients reported to have the habit of smoking. Patients were divided into two age groups: 839 (54.9%) patients were aged less than 60 years and 688 (45.1%) were aged 60 and above. Diagnostic angiograms were only completed for 955 (62.5%) patients, while 572 (37.5%) underwent PCI. Patient data are summarized in Table 1. 

**Table 1:** Baseline characteristics of patients The data are presented in the form of frequency (n) and percentage (%).

Age (Mean ± SD)	58.09 ± 8.07
Female n(%)	580 (38%)
Body Mass Index (Mean ± SD)	26.7 ± 4.3
Cardiovascular Risk Factors	
Hypertension n(%)	1236 (81%)
Dyslipidaemia n(%)	320 (21%)
Diabetes Mellitus n(%)	363 (23.8%)
Current Smoking n(%)	290 (19%)
Previous MI n(%)	626 (41%)
Medication Use	
Aspirin n(%)	1482 (97.1%)
Thienopyridine n(%)	1354 (88.7%)
Statins n(%)	1114 (73%)
Beta Blockers n(%)	1498 (97.1%)
ACE Inhibitors n(%)	1192 (78.1%)
ARBs n(%)	250 (16.4%)
Nitrates n(%)	1439 (94.3%)
Ranolazine n(%)	658 (43.1%)

Overall, the onset of RAO was found to be only in 81 (5.3%) patients. Pearson correlation tests and chi-square tests were performed to find the association of the onset of RAO with age, gender, medical procedure, and comorbidities. There was a significantly higher RAO rate in patients aged over 60 years (60.5% vs 39.5%, p = 0.003). There were also higher RAO rates with PCI compared to angiography (53.1% vs 46.9%, p = 0.071) and female compared to male patients (45.7% vs 54.3%, p = 0.143); however, the effects of both these variables were not significant (Table 2).

**Table 2:** Association of radial artery occlusion (RAO) with demographic variables

Variables	RAO n(%)	p-value	Chi-square value
Age			
< 60 years	32 (39.5%)	0.003	8.235
> 60 years	49 (60.5%)		
Gender			
Male	44 (54.3%)	0.143	2.151
Female	37 (45.7%)		
Medical Procedure			
PCI	38 (46.9%)	0.071	3.264
Angiography	43 (53.1%)		
Diabetes Mellitus			
Yes	20(24.7%)	0.912	0.12
No	61(75.3%)		
Hypertension			
Yes	69(85.2%)	0.233	1.424
No	12 (14.8%)		

## Discussion

In this study, the aim was to identify the onset rate and risk factors of RAO. The general rate of RAO onset was found to be 5.3%. Older patients (60 years and above ) were much more likely to have RAO than younger patients. Additionally, no significant association was found between RAO, gender, and high blood pressure. A previous study was conducted at the Ch. Pervaiz Elahi Institute of Cardiology in Multan included 180 patients who underwent TRA. The study group's age range was 18-70 years. Of the patients, 14 (7.8%) had RAO. There was no significant variation in RAO between age groups and genders [8]. Another study with 125 patients was conducted in Multan; results showed that the average age of patients was 65.22 ± 11.54 years. The patients' average body mass index was 29.93 ± 4.87 kg/m^2^. After 24 hours of surgery, five (4.0%) individuals presented with RAO [9]. The findings are similar to those of the current study, but the sample size of these studies was relatively small. In a prospective trial following TRA, 563 patients with a normal Allen test were assessed for radial artery patency and function using physical and ultrasound examinations at discharge and a one-month follow-up. Thirty patients (5.3%) had clinical signs of RAO at discharge. Sixteen patients (2.8%) had chronic RAO at follow-up. The study found that the risk factors of RAO following TRA were minimal [10]. In another study, there were 7,215 TRI patients, of which 68 (0.95%) had acute RAO. In the RAO group, there were more female patients with diabetes mellitus than in the standard group [11]. This highly variable rate of RAO is due to the difference in patient demographics, the methods used to achieve hemostasis, and the method used to assess radial artery patency after the TRA. Most studies estimate that early RAO occurs in less than 5% of cases, and the estimated incidence of RAO is 5-10%. Most studies check the pulse to assess radial artery patency before hospital discharge. In the United States, 20% will determine RAO incidence with echo-Doppler or oximetry/plethysmography testing [12,13].

TRA is a convenient option for patients with low rates of radial artery occlusion when performed by experienced operators. The use of technology can further improve outcomes.

Limitations

This was a single-center study, and patients with previous radial artery procedures were excluded. All patients included in the study were undergoing elective procedures; therefore, patients needing emergency catheterization procedures were excluded. Furthermore, echo-Doppler ultrasound can be used for radial artery assessment. This ultrasound can better define the artery and help in cannulation and assessment of RAO, which was not used in this study, because of the clinical gadget limitations. In addition, patients who switched to TFA and those who did not have the sheath removed immediately after the procedure were not studied.

## Conclusions

In summary, the incidence of RAO, following transradial percutaneous interventions, was shown to be minimal among the selected patient population. Notably, age was found to be a significant risk factor, with patients 60 years of age and older having a higher incidence of RAO. Gender, comorbidities, and the particular operation type (angiography or PCI) did not show any discernible correlations with the incidence of RAO. More research is necessary in diverse Pakistani locations to confirm these results and guarantee their generalizability to larger populations. These initiatives will help us understand RAO risk factors better and improve TRA procedure prevention tactics.
